# Activity of *Trichoderma asperellum* Strain ICC 012 and *Trichoderma gamsii* Strain ICC 080 Toward Diseases of Esca Complex and Associated Pathogens

**DOI:** 10.3389/fmicb.2021.813410

**Published:** 2022-01-28

**Authors:** Stefano Di Marco, Elisa Giorgia Metruccio, Samuele Moretti, Marco Nocentini, Giuseppe Carella, Andrea Pacetti, Enrico Battiston, Fabio Osti, Laura Mugnai

**Affiliations:** ^1^Institute of BioEconomy, National Research Council, Bologna, Italy; ^2^Plant Pathology and Entomology Section, Department of Agricultural, Food, Environmental and Forestry Science and Technology (DAGRI), University of Florence, Florence, Italy

**Keywords:** biocontrol agent, biological control, Esca complex, grapevine trunk diseases, *Trichoderma*

## Abstract

Grapevine trunk diseases are widespread in all grape-growing countries. The diseases included in the Esca complex of diseases are particularly common in European vineyards. Their distinctive foliar symptoms are well known to be associated not only with losses in quantity, as with all grapevine wood diseases, but also with losses in the quality of the crop. Protection of pruning wounds is known to reduce infections in artificial inoculations and, to some extent, reduce the external leaf symptoms. The application of biological control agents in the field is typically started at the first appearance of symptoms. In this article, the two strains belonging to two different species, *Trichoderma asperellum* ICC 012 and *T. gamsii* ICC 080, which are present in a commercial formulation, were tested *in vitro*, *in vivo* in artificial inoculation, and in the field in long-term experiments where the wounds on four young asymptomatic vineyards were protected since 1 or 2 years after planting. The *in vitro* trials highlighted the different temperature requirements of the two strains, the direct mycoparasitizing activity of *T. asperellum*, and the indirect activity shown by both *Trichoderma* strains. The *in vivo* trials confirmed the ability of the two strains to reduce the colonization following artificial inoculations with the high, unnatural concentration of spores used in artificial infections, even if with variable efficacy, and with long persistence as they could be reisolated 7 months post-application. The preventive applications carried out over 9 years showed a very high reduction in symptom development in the treated vines, on annual and cumulated incidence and on the death of vines, with disease reduction varying from 66 to almost 90%. Early and annual application of protection to the pruning wounds appears to be the best method for reducing damages caused by grapevine leaf stripe disease (a disease of the Esca complex of diseases). *Trichoderma* appears to offer an efficient, environmentally friendly, and long-lasting protection in the presence of a natural inoculum concentration.

## Introduction

Grapevine trunk diseases (GTD), in recent decades, have shown an increasing impact on grapevine cultivation, causing severe losses in viticulture (Mondello et al., 2018a). The spread of the diseases is due to multiple interacting factors, such as the dissemination of potentially infected propagation material and, above all, the adoption of methods of production in vineyards that favored fungal infections or weakened the vine defense mechanisms (Surico et al., 2004; Gramaje and Di Marco, 2015; Lecomte et al., 2018). Therefore, the increasing spread of GTDs in almost all grape-growing areas and the onset of symptoms, even on young vines, have attracted the attention of winegrowers and researchers on such diseases.

The most relevant and damaging GTD in Europe was commonly known as “Esca disease” of grapevine (Larignon and Dubos, 1997; Bertsch et al., 2013; Guerin-Dubrana et al., 2019) but has been re-defined by some authors, based on research carried out in the recent decades, as a complex of different diseases (Mugnai et al., 1999; Surico, 2009), where grapevine leaf stripe disease (GLSD), without or with wood decay (Esca proper), is characterized by the typical leaf stripe symptoms.

Tracheomycotic pathogens, like *Phaeomoniella chlamydospora* (*Pch*) and *Phaeoacremonium* species [mainly *Phaeoacremonium minimum* (*Pmin*)], and the basidiomycete *Fomitiporia mediterranea* (*Fmed*) are commonly considered as the main pathogens associated with Esca complex of diseases (ECDs), inducing wood discoloration and decay, respectively. Moreover, fungal canker agents, like botryosphaeriaceous species, are very often isolated from affected vines (Bertsch et al., 2013; Gramaje et al., 2018).

**FIGURE 1:**
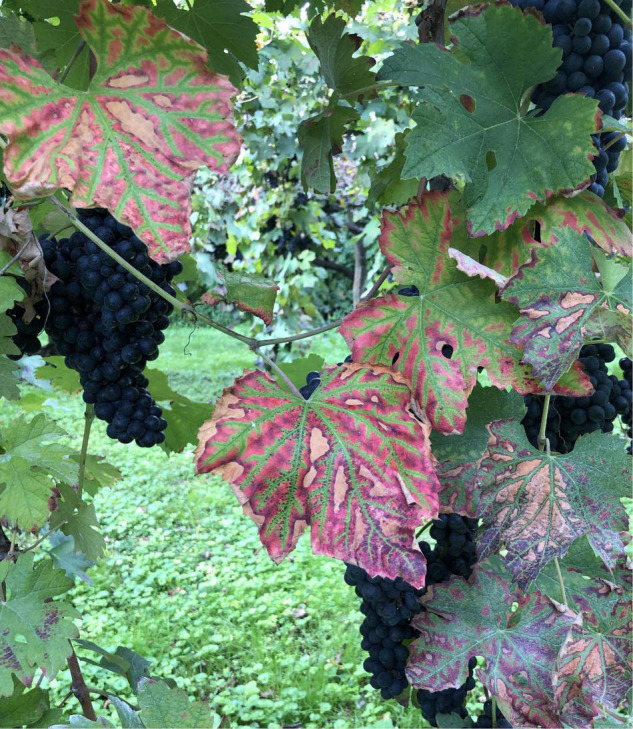
Leaf stripe symptoms, often described as tiger stripe leaves, are the most distinctive symptom of grapevine leaf stripe disease, within the Esca complex of diseases.

The infected plants can show various types of symptoms in the canopy (among which the leaf stripe symptom is one of the most characteristic feature; Figure 1) and in the wood (wood rot and central and sectorial discolorations and necrosis) (Mugnai et al., 1999). The role of various pathogens associated to leaf symptoms has not been clarified completely yet, and the relationship with white rot presence and extent has recently been reviewed (Moretti et al., 2021). The leaf symptoms have been reproduced only on very few occasions by the inoculation of different pathogens (Sparapano et al., 2001; Reis et al., 2016; Markakis et al., 2017). GLSD appears not to be linked to specific fungal agents but as a reaction of the plant to the activity of fungal wood colonization affecting the plant physiology and the quality of production (Calzarano et al., 2004b,2017a; Lorrain et al., 2012; Pouzoulet et al., 2014; Fontaine et al., 2016; Mary et al., 2017; Bortolami et al., 2021; Del Frari et al., 2021).

Foliar symptom expression shows almost unique peculiarities, as their intermittent occurrence in the vineyard (Marchi et al., 2006; Guerin-Dubrana et al., 2019) is a very rare phenomenon that has been observed in another wood disease, the “decay of kiwi fruit” that has many common factors with ECDs (Di Marco and Osti, 2008). The erratic nature of foliar symptom expression could be associated with phytotoxins produced by tracheomycotic pathogens, alterations of phytoalexins and photosynthetic functions, and disorders of sap flow or hydraulic failures (Claverie et al., 2020; Calzarano et al., 2021; Ouadi et al., 2021) modulated by environmental and meteorological factors, such as rainfall and temperature (Marchi et al., 2006; Calzarano et al., 2018). Nevertheless, the deterioration of the wood by pathogens and the correlation between incidence and severity of foliar symptom expression and yield losses are unquestionable. In that respect, ECDs control aims to reduce the risk and effects of wood infection (Mondello et al., 2018a).

Management of infected vines includes different approaches like trunk renewal (Egger et al., 1998; Calzarano et al., 2004a; Gramaje et al., 2018), wood surgery or curettage (Bruez et al., 2020; Pacetti et al., 2021), vine training for an optimal sap flux (Lecomte et al., 2018), or re-grafting of infected plants in the vineyard as alternative to replanting (Mondello et al., 2018a). Foliar treatments can also reduce disease incidence as (i) with fosetyl Al application in a downy mildew strategy (Di Marco et al., 2011a), (ii) a nutrient mixture based on calcium, magnesium, and seaweed (Calzarano et al., 2007, 2017b; Calzarano and Di Marco, 2018), or (iii) even winter treatments with a copper formulation (Di Marco et al., 2011b; Calzarano et al., 2014). Nevertheless, the best way to control wood pathogens remains to be reduction of the risk of infection, and many efforts have been focused on the protection of pruning wounds, as pathogens, mostly spread by airborne spores, can cause new infections mainly through the pruning wounds (Mugnai et al., 1999; Bertsch et al., 2013).

Pruning wounds are susceptible to infections for a long time, at least up to 4 months (Eskalen et al., 2007; Serra et al., 2008). The susceptibility of wounds is linked to many factors, such as (i) age of the wound at the time of infection (Eskalen et al., 2007), (ii) cultivar (Feliciano et al., 2004; Mutawila et al., 2011a), (iii) type and/or time of pruning (Rolshausen et al., 2010; Elena and Luque, 2016), and (iv) climatic conditions, such as frequency of rain events (Eskalen and Gubler, 2001; Rolshausen et al., 2010) or weekly temperatures (Serra et al., 2008). The protection of pruning wounds from GTDs has been achieved by application of different fungicides (Mondello et al., 2018b) as well as by applying new specific formulations as a paste (Diaz et al., 2013). A new product based on a polymer and fungicides and known as pyraclostrobin + boscalid (Tessior^®^), applied in the vineyard with special equipment on each pruning wound, was proposed against ECDs and registered in some European countries (Lengyel et al., 2019).

The long susceptibility of pruning wounds to infections and the need for long-lasting colonization and activity, combined with the need for sustainable tools, promoted the setting up of wound protection with biological control agents, such as *Trichoderma* species (Di Marco et al., 2004; Van Niekerk et al., 2011; Bigot et al., 2020; Brown et al., 2021) and *Bacillus* spp. (Alfonzo et al., 2012; Mondello et al., 2018a).

The *Trichoderma* species are filamentous ascomycetes usually living in the root and soil ecosystem and widely applied as bio-control agents. The bio-control is due to various antimicrobial activities, including mycoparasitism, antibiosis, competition for nutrients and/or space, and induction of plant resistance (Harman et al., 2004). Research on the use of *Trichoderma* toward GTDs was primarily performed in the nursery during the process of production of propagation material with applications in hydration, grafting, callusing, and nursery field (Halleen et al., 2001; Hunt et al., 2001; Di Marco et al., 2002, 2004; Fourie and Halleen, 2004, 2006; Di Marco and Osti, 2007; Mounier et al., 2016; Pertot et al., 2016).

Pruning wound protection was initially performed with strains of *Trichoderma longibrachiatum* (strain 6) or *Trichoderma harzianum* (strain T39) that were able to colonize the pruning wound surface for up to 2 months in the vineyard, while applications to pruning wounds on potted vines before artificial inoculation with *Pch* prevented necrosis in xylem tissue below the wound (Di Marco et al., 2004). Other studies showed the activity of *Trichoderma* species in protecting pruning wounds from infections by different GTD pathogens, including *Pch* (Kotze et al., 2011). Further research pointed out that the activity of *Trichoderma* species is also dependent on the interaction with the host-plant cultivar (Mutawila et al., 2011a). The growth and interaction between *T. harzianum*, *Eutypa lata*, and *Pch* showed that the bio-control agent was able to grow deeper in the presence of the pathogens than when applied alone on the wound (Mutawila et al., 2011b). *Trichoderma atroviride* strain SC1 colonized the wounds for several months after its application, protecting the plants throughout the growth season (Prodorutti et al., 2012). Several *Trichoderma*-based products were registered against ECDs. *Trichoderma atroviride* I-1237 (Esquive^®^) was demonstrated to be effective in wound protection against “Esca”, black dead arm, and *Eutypa* dieback, reducing leaf symptom expression after at least a 2-year application (Mounier et al., 2016) and reducing the infections on pruned canes artificially inoculated with *Pch* and *Neofusicoccum parvum* (Reis et al., 2017). *Trichoderma atroviride* SC1 (Vintec^®^) reduced the infections when applied in the nursery and in the vineyard (Berbegal et al., 2020). Some activity of *T. atroviride* SC1 and *T. atroviride* I-1237, even if significantly lower than that of new synthetic fungicide formulations, was observed on wounds artificially infected with *Pch* and *Diplodia seriata* (Martinez-Diz et al., 2021). Decreased incidence and severity of foliar symptoms of Esca were obtained in vineyard pruning wounds treated with Remedier^®^, a bio-fungicide composed of *Trichoderma asperellum* ICC 012 and *Trichoderma gamsii* ICC 080 in a formulation developed by Isagro Company (Aloi et al., 2017; Bigot et al., 2020).

This study aimed to characterize the protective activity of a *Trichoderma*-based commercial product, Remedier^®^, when applied in a really preventive approach in yet asymptomatic vineyards, demonstrating that its constant use over a long length of time gives an increasing efficacy as a strong reduction agent of ECDs symptom development. The *in vitro* activity of the two individual strains at different temperatures was assessed against three GTD pathogens (*Pch*, as the main tracheomycotic pathogen of ECDs; *N. parvum*, as an important canker agent; and the white-rot agent *F. mediterranea*). In the vineyard, the activity of the registered formulation Remedier^®^ and the viability of both *Trichoderma* species after different times of application were assessed for their efficacy to protect pruning wounds inoculated with *Pch*. Finally, the effect of a field spray application of Remedier^®^ was evaluated on the annual and cumulative leaf stripe symptoms incidence and the percentage of apoplexy and mortality over a multiple-year survey carried out in four vineyards located in two different regions of Italy that were treated to protect them from infections since after 1 or 2 years from planting.

## Materials and Methods

### *In vitro* Growth of *Trichoderma* Strains at Different Temperatures

Isagro S.p.A (Novara, Italy) provided fungal colonies of *T. asperellum* strain ICC 012 (*Tasp*) and *T. gamsii* strain ICC 080 (*Tgam*), which are the active ingredients of the commercial bio-fungicide Remedier^®^. *Trichoderma* colonies were grown on marine agar (MA; Difco, Detroit, MI, United States) in Petri dishes and maintained at 25 ± 1°C with a 12-h photoperiod. Plates of MA were inoculated by placing a plug of *Tasp* or *Tgam* face down at the center of the dish. The plates were then incubated at 10, 13, 18, and 23 ± 1°C with a 12-h photoperiod, and colony radii were recorded daily. There were five replicates for each *Trichoderma* strain–temperature combination. Measurements of daily growth of the two strains at the different temperatures were continued until the colonies reached the edge of the dish or stopped growing.

### *In vitro* Activity of the Two *Trichoderma* Strains Against Selected Wood Pathogens at Different Temperatures

Each *Trichoderma* strain was tested against *Pch* CBS229.95, *Fmed* strain PVFi-201.03, and *N. parvum* (*Np*) PVFi-np22 (maintained in the collection of DAGRI, University of Florence, Florence, Italy). All fungal colonies were grown under the conditions described above.

A mycelial plug (5 mm in diameter) of a colony of *Tasp* or *Tgam* was put face down at the periphery of a Petri dish, and a colonized agar plug of each pathogen isolate was placed at the opposite side along the diameter of the same plate. Plates containing only a plug of each *Trichoderma* strain or pathogen strain served as control. The plates were incubated at 10, 13, 18, and 23 ± 1°C with a 12-h photoperiod, and colony radii along the diameter were measured daily until the *Trichoderma* strain and pathogen colonies in the same plate stopped growing. There were five replicates for each *Trichoderma*–pathogen–temperature combination.

The direct (competitive growth) or indirect (production of volatile and non-volatile compounds) activity of the *Trichoderma* strains was assessed as follows:

Direct activity of *Trichoderma* was recorded as (i) the day when the *Trichoderma* strain and pathogen colonies came into contact, (ii) the day when maximum overgrowth, the ability of the *Trichoderma* strain to overlap and grow over the pathogen colony, occurred and at what percentage, and (iii) the day when the *Trichoderma* strain sporulated on the pathogen colony. At the maximum *Trichoderma* overgrowth, the colony area of the pathogen and the area overlapped by *Trichoderma* were measured by video image analysis. The images were taken with a CCD camera (model TK-880, JVC, Yokohama, Japan) interfaced with a computer by an ELVIS board and Chameleon software (Sky Instruments Ltd., Llandrindod Wells, Powys, Wales, United Kingdom). The percentage of maximum overgrowth was calculated by dividing the overlapped colony area by the colony area multiplied by 100.

Indirect activity of *Trichoderma* was recorded as (i) the day when the pathogen colony stopped growing and (ii) the day and the percentage of maximum colony growth inhibition assessed on the day immediately before the contact between the *Trichoderma* strain tested and the pathogen. In case there was no contact, the day when both the *Trichoderma* strain and pathogen stopped growing was recorded. Inhibition of the pathogen colony at each temperature was calculated as follows: radius of the pathogen colony without *Trichoderma* minus radius of the pathogen colony in the presence of *Trichoderma* divided by the radius of the pathogen colony without *Trichoderma* multiplied by 100. Both radii of pathogen colonies grown with or without *Trichoderma* were assessed on the same day.

### Artificial Inoculations on Grapevine Canes Treated With the *Trichoderma* Formulation in an In-Field Trial

All field trials were carried out with the commercial product Remedier containing^®^ 2% *T. asperellum* strain ICC 012 (*Tasp*) and 2% of *T. gamsii* strain ICC 080 (*Tgam*). As a parallel test, inoculation of canes previously protected by spraying the commercial product Remedier^®^ was carried out. The same number of control canes were included in all tests. In the untreated control canes, no protection nor artificial inoculation was applied. The trial was carried out in a vineyard of 20-year-old “Sangiovese” grapevines located in San Casciano in Val di Pesa, Florence, Italy, where vine rows where no ECDs foliar symptoms had been recorded in the previous 3 years were selected. In 2009 and 2010, at BBCH growth stage 00 (winter buds), canes on the cordon that were as much as possible perpendicular to the soil were selected. The canes were pre-pruned at the end of February, and a few centimeters were cut from the tip of the pruned cane to provide a fresh wound on the day when the treatment was applied. Following the recommendation of the producer, the commercial *Trichoderma* formulation was prepared by pouring the product into water at room temperature at about 24 h before the application so as to start the germination of the spores. On the following day, the treatment was applied by a hand sprayer at the label concentration (250 g hL^–1^) up to dripping. At 4 days after treatment, 100 μL of a 1 × 10^5^ *Pch* mL^–1^ conidial suspension was applied to the wound of each cane.

Forty-five canes were used per treatment (three replicates of 15 canes each). After 7 months, re-isolations were made from the canes. The canes were quickly soaked in ethanol and flamed, the bark was removed, and the canes were flamed again after spraying with ethanol. Three 2-mm-thick wood disks or transversal sections were sampled from each cane at 0.5, 2, and 6 cm away from the cut and inoculated end. Moreover, the first external slice was also analyzed to assess the survival of the *Trichoderma* species. Each slice, 2 mm in thickness, was cut in six fragments that were plated on MA agar. The colonies that developed in the following 60 days were identified from their morphology (characteristics of the colony and micromorphology).

### Colonization and Persistence of the *Trichoderma* Formulation on Artificially Inoculated Canes

The colonization ability and persistence of the two strains of *Tasp* and *Tgam* present in the commercial product were assessed in a further trial setup in 2010 in the same vineyard in San Casciano in Val di Pesa (Florence). A further lot of 90 canes was treated with Remedier^®^ immediately after the trimming cut as detailed above. The same number of canes was left unprotected as untreated control to check for possible natural *Trichoderma* infections. Re-isolation was carried out by sampling 15 canes at 3, 7, and 15 days (to assess establishment speed) and 1, 2, and 7 months after the spray application (to assess persistence over time). In both trials, 6 wood chips per cane, taken from the first 5 mm of the cut end in each trimming cut, were plated on MA. Colonization and persistence were evaluated as the number of canes with at least one colony (incidence) and as the total number of wood chips colonized by each of the two *Trichoderma* species (severity).

### Efficacy of Application of the *Trichoderma*-Based Product in the Vineyard

The field trials details are shown in Table 1. The vineyards, Guyot trained, were located in two regions of Central and Northern Italy: Emilia-Romagna and Friuli Venezia Giulia. In each vineyard, at the year before the start of the experiments (September), a time 0 survey was carried out to assess the absence of ECDs symptoms. In the following spring (March), two plots were selected in each vineyard: one was treated with the *Trichoderma* commercial formulation, and the other one was used as untreated control. In each vineyard, around 500 vines per plot (2 plots per vineyard) were monitored each year and mapped (Table 1).

**TABLE 1:** List of the vineyards where the on-field applications of the *Trichoderm*a formulations on pruning wounds were carried out.

Vineyard code and cultivar	Region	Locality, province	Year of planting	Time of pruning	Soil	*Trichoderma* application			Number of plants per plot at the beginning of the trial	
						First	Last	Time	Control	*Trichoderma*-treated
V1 Trebbiano	Emilia-Romagna	S. Andrea, Ravenna	2009	Mid-February	Medium texture	2011	2019	Early April	488	486
V2 Lambrusco	Emilia-Romagna	Cavezzo, Modena	2009	Mid-March	Medium texture	2011	2019	Early April	799	800
V3 Trebbiano	Emilia-Romagna	Bagnara, Ravenna	2009	Mid-February	Medium texture	2011	2018	Early April	362	384
V4 Cabernet Franc	Friuli Venezia Giulia	Tauriano, Pordenone	2009	Mid-March	Medium texture	2010	2019	Late March	600	600
										

*Trichoderma* applications were carried out every year with the same modality for the following 9 years. The trials were conducted in commercial vineyards planted in the previous year. Except for the wound protection treatment, both plots in each vineyard received the same treatments against pests and foliar diseases.

As mentioned above, the commercial formulation of *Trichoderma* was prepared 24 h before its application to start the germination of the spores. The product was applied at 250 g hL^–1^ formulation, with a volume of 400 L ha^–1^, thus distributing 1 kg of product per hectare. A single application per year was carried out in late March until early April.

Every year, the vineyards were monitored at the end of August to early September, time of greatest disease expression. The position of each plant in the plot and its condition and symptoms were recorded each year, following an arbitrary scale of symptom severity on a two-dimensional map to follow the evolution of the disease on a single plant over the years of investigation, as healthy (“H”) or with low severity (“A”) of foliar symptoms and high severity (“B”) of foliar symptoms when expressing 5–30% or >30% symptoms of the whole vine canopy.

The frequency of occurrence of the two levels of symptoms (A or B) was calculated by dividing the total number of vines with A or B symptom level in a given year by the total number of standing plants of the same age in that year multiplied by 100. Furthermore, the frequency of plants showing apoplexy (APO), as wilting of foliage and clusters of the whole plant, was calculated by dividing the number of plants showing symptoms of apoplexy in a given year by the total standing coetaneous plants in that year multiplied by 100.

The annual incidence was calculated by dividing the number of plants with leaf symptoms in a given year by the total number of standing coetaneous plants (H + A + B + APO) in that year multiplied by 100. The cumulated incidence was calculated in each year of survey by dividing the standing plants with leaf symptoms in that year or in at least one of the previous years of survey by the total number of standing coetaneous plants observed in the first year of survey multiplied by 100. The cumulated number of dead plants was calculated each year of the survey by dividing the total number of plants that died after showing foliar symptoms or apoplectic symptoms in that or in a previous year of investigation by the total number of standing coetaneous plants in the first year of survey and multiplying by 100.

In each year, the efficacy of the protective action was calculated as the percentage of vines that had been symptomatic at least once during the survey in the treated or untreated plots (cumulated incidence) over the total number of vines that had been symptomatic at least once over the years (cumulated incidence) in each vineyard.

### Statistical Analyses

#### *In vitro* Trials

For each pathogen, analysis of variance (ANOVA) was used to determine the significance of the differences between the treatments in the daily mycelium growth rate of the two *Trichoderma* strains and their inhibition activity against pathogens *in vitro*. In the case of indirect activity, results were submitted to ANOVA, and Tukey’s test was used to assess the difference among treatments (*P* = 0.05). Statistical analyses were performed using SAS system software, version 9.1 (SAS Institute, Cary, NC, United States).

### Artificial Inoculations on Grapevine Canes Treated With the *Trichoderma* Formulation in an In-Field Trial

Data normality was checked with the Shapiro–Wilk test. The data were analyzed statistically using Kruskal–Wallis non-parametric and Dunn’s *post hoc* tests; the significance values were adjusted by Bonferroni correction *via* SPSS 27 software (SPSS Inc., Chicago, IL, United States). The statistical significance level was set at *P* = 0.05.

#### Application of the *Trichoderma*-Based Product in the Vineyard

The activity of *Trichoderma* on annual disease incidence, cumulated disease incidence, symptom A and symptom B incidence, apoplexy incidence, and cumulated numbers of dead plants were analyzed by χ^2^ test. All the plants of each plot were considered to compare *Trichoderma* and control treatments. Where differences in distribution between the two treatments were significant, asterisks were placed (*P < 0.05, *P* < 0.01, and ^***^P < 0.001 significance levels, respectively). Statistical analyses were performed using Prism 5 (GraphPad Software, San Diego, CA, United States). The reduction in the cumulative percentages of symptomatic vines was calculated using Abbott’s formula (Abbott, 1925) for comparison among treatments and untreated control.

## Results

### *In vitro* Growth of *Trichoderma* Strains at Different Temperatures

**FIGURE 2:**
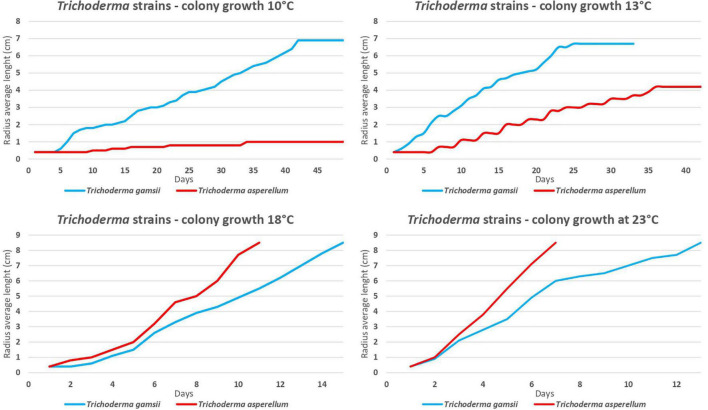
*Trichoderma asperellum* strain ICC 012 (*Tasp*) and *T. gamsii* strain ICC 080 (*Tgam*) growth at 10, 13, 18, and 23°C on marine agar.

*Trichoderma asperellum* strain ICC 012 (*Tasp*) and *T. gamsii* strain ICC 080 (*Tgam*) showed different growth rates at different temperatures. *Tasp* had a higher radius length (*P* < 0.05) at 18°C from the 4th day after the beginning of the trial and at 23°C from the 3rd day compared to *Tgam.* The radius length of *Tgam* increased faster (*P* < 0.05) than *Tasp* at 10 and 13°C from the 5th and 2nd day from the beginning of the trial (Figure 2). On the whole, *Tasp* grew faster at higher temperatures, while *Tgam* grew faster at lower temperatures.

### *In vitro* Activity of *Trichoderma* Species Against Selected Wood Pathogens at Different Temperatures

The pathogens grew at 13, 18, and 23°C. *Np* grew also at 10°C, while the other pathogens did not grow beyond 1 mm.

#### Direct Activity

*Tasp* overgrew the pathogen colonies at 18 and 23°C. At 23°C, *Tasp* was significantly (*P* < 0.05) faster than at 18°C in reaching the pathogen colony of *Pch* and displayed maximum overgrowth after 2–5 days from contact with the colonies of the pathogen. At both temperatures, overgrowth was complete (100%) for *Pch* and *Fmed* and partial for *Np*, where the percentage of overgrowth at 23°C was significantly (*P* < 0.05) faster than at 18°C (Table 2).

**TABLE 2:** *In vitro* direct activity of the *Trichoderma asperellum* strain (*Tasp*) on *Phaeomoniella chlamydospora*, *Neofusicoccum parvum*, and *Fomitiporia mediterranea*.

Temperature (°C)	*Phaeomoniella chlamydospora*				*Fomitiporia mediterranea*				*Neofusicoccum parvum*			
	Contact	Maximum overgrowth		Sporulation	Contact	Maximum overgrowth		Sporulation	Contact	Maximum overgrowth		Sporulation
	Day^a^	Day^a^	%^a^	Day^a^	Day	Day	%	Day		Day	%	Day
10	–^b^	–^b^	–^b^	–	–	–	–	–	–	–	–	–
13	–	–	–	–	37a^c^	–	–	–	20a	–	–	–
18	10a	12a	100°	12°	7b	11a	100a	11a	6b	10a	28.2a	14a
23	4b	9a	100°	6b	7b	9a	100a	9a	4b	6b	42b	9b

*^a^Days after plug deposition in the plate.*

*^b^For each pathogen, the dash within a column indicated that no contact, overgrowth, or sporulation occurred, and statistical analysis was not performed.*

*^c^For each pathogen, data followed by the same letter within columns did not differ significantly according to ANOVA (P < 0.05).*

*Tasp* sporulated earlier at 23°C than at 18°C (significantly different at *P* < 0.05 for *Pch* and *Np*). In the interaction with *Np*, *Tasp* sporulation was less efficient compared to *Pch* and *Fmed*, occurring 4 days after the maximum overgrowth. The maximum overgrowth percentage ranged from 28% at 18°C to 42% at 23°C, and the values were significantly different (*P* < 0.05, Table 2). *Tgam* was not able to overgrow the pathogens.

#### Indirect Activity

In general, *Tasp* did not reduce the growth of the pathogens except for *Np* at 13°C, where growth was reduced by 48%, and on *Fmed*, where growth was reduced by 52% (Table 3). In contrast, *Tgam* stopped and reduced the growth of all pathogens at all temperatures at which pathogens grew. Pathogen colonies were stopped more rapidly at higher temperatures. The percentage of *Np* colony growth inhibition was significantly (*P* < 0.05) higher (63.5%) at 10°C, whereas the greatest inhibition occurred at 18 and 23°C on *Fmed* (65%) and *Pch* (55.5%), respectively (Table 3).

**TABLE 3:** Indirect activity of *T. asperellum* (*Tasp*) and *T. gamsii* (*Tgam*) strains in stopping or reducing the growth of the pathogen colony in dual culture with *Phaeomoniella chlamydospora* (*Pch*), *Neofusicoccum parvum* (*Np*), and *Fomitiporia mediterranea* (*Fmed*).

Pathogen	Temperature	Growth stop	Highest growth inhibition^b^	
	(°C)	Day^a^	Day^a^	(%)

***Tasp* activity on pathogen growth**				
*Pch*	10	–^c^	–	–
	13	–	–	–
	18	–	–	–
	23	–	–	–
*Np*	10	–	–	–
	13	6	18	48
	18	–	–	–
	23	–	–	–
*Fmed*	10	–	–	–
	13	23	34	52
	18	–	–	–
	23	–	–	–

***Tgam* activity on pathogen growth**				

*Pch*	10	–^c^	–	–
	13	9	15	8.7c^d^
	18	9	9	24b
	23	9	15	55.5a
*Np*	10	14	24	63.5a
	13	8	10	46b
	18	6	8	15c
	23	4	6	38b
*Fmed*	10	–	–	–
	13	19	42	17a
	18	10	16	65b
	23	6	10	61b

*^a^Days of growth in Petri plates.*

*^b^The reduction was assessed immediately before the contact between Trichoderma and pathogen colonies.*

*^c^The colony of the pathogen did not grow.*

*^d^For each pathogen, data followed by the same letter within columns did not differ significantly according to Turkey’s test (P ≤ 0.05).*

### Artificial Inoculations on Grapevine Canes Treated With the *Trichoderma* Formulation in an In-Field Trial

The commercial *Trichoderma* formulation significantly reduced (*P* = 0.05) the colonization by *Pch* in the first-year trial in 2009 when it almost completely stopped pathogen invasion in canes at 0.5, 2, and 6 cm from the wound, as can be seen compared with the positive control, i.e., inoculated but non-protected canes (Table 4—2009). In the same trial, *Trichoderma* was still present and re-isolated from 24% of the sampled wood fragments. In the following year, the results were quite different, as the colonization by the pathogen was quite high in the first analyzed section, at 0.5%, reaching a high severity (Table 4—2010). Nevertheless, the pathogen, even if present in the first section, was reduced at 2- and 6-cm distance from the wound. The number of canes with at least one infection was reduced to 31% of the canes. *Trichoderma* was re-isolated from 87% of the control-treated canes.

**TABLE 4:** Colonization of canes by *Phaeomoniella chlamydospora* (*Pch*) and *Trichoderma* species following preventive treatment by the *Trichoderma* formulation and/or artificial inoculation with *Pch*.

Cane wood analyzed	Untreated, non-inoculated	Untreated, inoculated (*Pch*)	Treated (*Trichoderma*), non-inoculated	Treated (*Trichoderma*), inoculated (*Pch*)
**2009**				
Whole cane^a^ (*Pch*)	0.6 ± 2.4*b*^b^	15.9 ± 14.9a	0.0 ± 0.0b	0.1 ± 0.8b
Section A^c^ (*Pch*)	0.0 ± 0.0b	12.6 ± 20.1a	0.0 ± 0.0b	0.0 ± 0.0b
Section B (*Pch*)	0.0 ± 0.0b	16.7 ± 21.9a	0.0 ± 0.0b	0.4 ± 2.5b
Section C (*Pch*)	1.8 ± 7.3b	18.5 ± 26.4a	0.0 ± 0.0b	0.0 ± 0.0b
Whole cane^a^ (*Trichoderma*)	0.1 ± 0.8b	0.4 ± 2.5b	24.6 ± 22.4a	20.1 ± 18.4a
Section A (*Trichoderma*)	0.4 ± 2.5b	0.7 ± 5.0b	43.0 ± 38.2a	31.1 ± 35.8a
Section B (*Trichoderma*)	0.0 ± 0.0b	0.0 ± 0.0b	21.8 ± 32.9a	17.0 ± 30.5a
Section C (*Trichoderma*)	0.0 ± 0.0b	0.4 ± 2.5b	8.9 ± 25.8ab	12.2 ± 24.7a
**2010**				
Whole cane^a^ (*Pch*)	0.4 ± 2.0*b*2	20.0 ± 19.3a	0.0 ± 0.0b	17.0 ± 13.6a
Section A (*Pch*)	1.1 ± 6.1b	36.1 ± 32.5a	0.0 ± 0.0b	46.7 ± 36.7a
Section B(*Pch*)	0.0 ± 0.0b	17.8 ± 28.3a	0.0 ± 0.0b	4.4 ± 9.7ab
Section C (*Pch*)	0.0 ± 0.0b	6.1 ± 18.8a	0.0 ± 0.0b	0.0 ± 0.0b
Whole cane^a^ (*Trichoderma*)	0.0 ± 0.0b	0.0 ± 0.0b	2.8 ± 6.6a	0.6 ± 1.7ab
Section A (*Trichoderma*)	0.0 ± 0.0b	0.0 ± 0.0b	6.1 ± 14.2a	1.1 ± 4.2ab
Section B (*Trichoderma*)	0.0 ± 0.0a	0.0 ± 0.0a	2.2 ± 12.2a	0.6 ± 3.0a
Section C (*Trichoderma*)	0.0 ± 0.0a	0.0 ± 0.0a	0.0 ± 0.0a	0.0 ± 0.0a

*The canes were pruned and treated in March 2009 and March 2010 in a cv Sangiovese vineyard. Re-isolation of the inoculated/applied fungi was carried out after 7 months in both years.*

*^a^In the whole cane, for each treatment, all the data from the three sections were averaged and statistically analyzed.*

*^b^Values are means ± standard deviation of three replicates, with 15 canes each. Values in the same row followed by different letters indicate significant differences based on Dunn’s test at p = 0.05.*

*^c^Sections A, B, and C are obtained at 0.5, 2, and 6 cm from the surface of the pruning wound, respectively.*

### Colonization and Persistence of the *Trichoderma* Formulation on Artificially Inoculated Canes

The ability of the two *Trichoderma* strains to colonize fresh pruning wounds was evaluated as the total number of wood chips colonized divided by the total number of sampled chips. Colonization was confirmed to be stable over time, and after 2 months from application, it was still present in up to 83.3% of the wood fragments plated. Even after 7 months, 60% of the wood fragments were still colonized by the *Trichoderma* strains. The *Tasp* strain, which is the one that is more efficient in direct activity against the wood pathogens tested, was more abundantly isolated in the first part of the season, while both strains were equally present on the wounds in the last part of the season (Table 5).

**TABLE 5:** Persistence of the colonization of fresh pruning wounds over time of the *Trichoderma* species applied as a commercial formulation (Remedier^®^).

Time after treatment	Wood chips analyzed	Wood chips with *Trichoderma* spp.		*Trichoderma* species re-isolation (%)		Average temperature (°C)	Average rain (mm)
	No.	No.	%	*% T. asperellum*	*% T. gamsii*		
**Colonization (100% of the inoculated canes at each sampling time and at least one colony of *Trichoderma*)**							

3 days	90	54	60.0	75	25	9	106.6
7 days	90	60	67.0	78	22	9	106.6
15 days	90	71	78.9	92	8	9	106.6

**Persistence (100% of the inoculated canes at each sampling time and at least one colony of *Trichoderma*)**							

1 month	90	81	90.0	50	40	9	106.6
2 months	90	75	83.3	53	30	14.2	11.8
7 months	90	54	60.0	53	47	17.8	17.6

*Re-isolation from the first 5 mm of the cut end in the pruning wound of 15 treated canes for each timing was analyzed after variable timing from application. Values are based on the total number of wood chips analyzed (six wood chips per cane, at 15 canes per treatment).*

### Application of *Trichoderma* Formulation in Young Asymptomatic Vineyards

**FIGURE 3:**
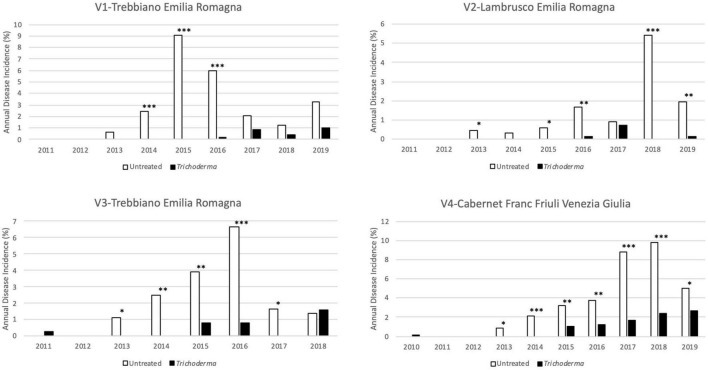
Annual disease incidence in the four young asymptomatic vineyards monitored for 8–10 years. Data were analyzed by χ^2^ test. All the plants of each plot were considered to compare *Trichoderma* and control treatments. Where differences in distribution between the two treatments were significant, asterisks were placed (*P < 0.05, ^**^P < 0.01, and ^***^P < 0.001 significance levels, respectively).

ECDs foliar symptoms appeared at 4–5 years after planting in the untreated plots. The *Trichoderma* treatments delayed the appearance of symptoms by 2 years (V3-Trebbiano and V4-Cabernet Franc) or 3 years (V1-Trebbiano and V2-Lambrusco). Furthermore, in all vineyards, since the first appearance of symptoms, a reduction in the percentage of symptomatic plants in the treated plots was clearly recorded (Figure 3).

**FIGURE 4:**
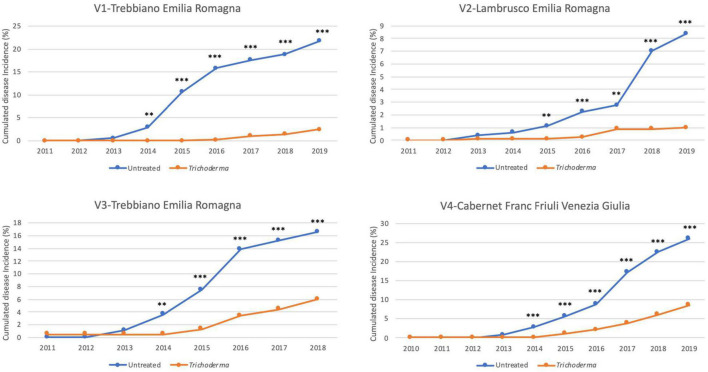
Yearly cumulated disease incidence in the four young asymptomatic vineyards monitored for 8–10 years. Data were analyzed by χ^2^ test. All the plants of each plot were considered to compare *Trichoderma* and control treatments. Where differences in distribution between the two treatments were significant, asterisks were placed (^**^P < 0.01 and ^***^P < 0.001 significance levels, respectively).

The reduction in the annual incidence was confirmed over the years from the onset of foliar symptoms and was significantly different in 80.8% of the year/vineyard combinations, 38.1% of which were at *P* < 0.001, 28.6% at *P* < 0.01, and 33.3% at *P* < 0.05% (Figure 3). The percentage of plants that showed symptoms in at least 1 year of the survey ranged from 8.4 to 26% in the untreated plot, whereas in the treated plots the percentage ranged from 1 to 8.7% (Figure 4). A statistically significant reduction of the cumulated incidence in the treated plot was observed the year after (2 years in V2-Lambrusco) the appearance of the foliar symptoms. This reduction was always significantly different (81.8% at *P* < 0.001 and 18.2% at *P* < 0.01) throughout the survey (Figure 4).

**FIGURE 5:**
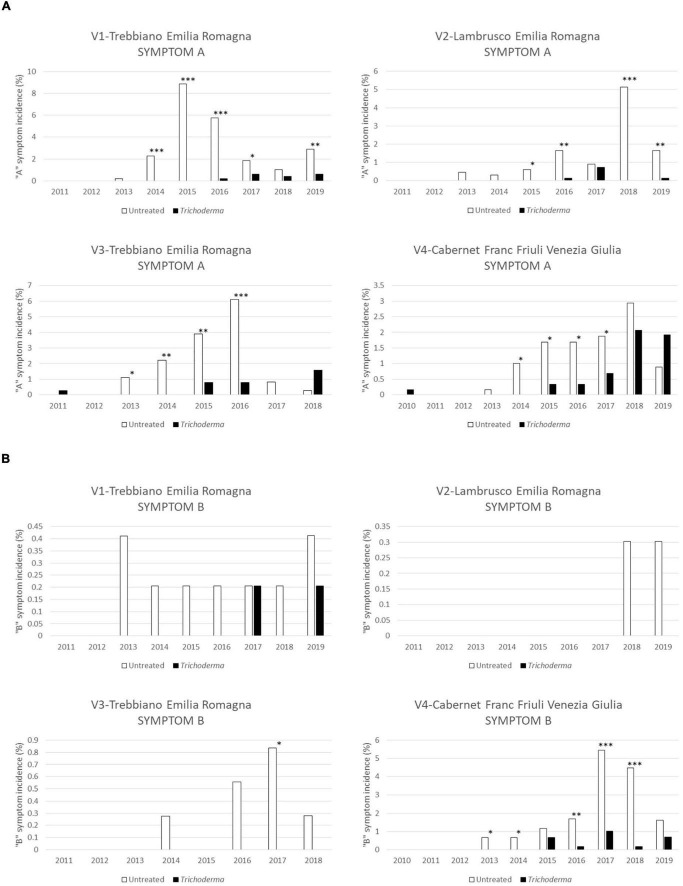
Severity of foliar symptoms in the four young asymptomatic vineyards monitored for 8–10 years: Symptom “A”—low severity **(A)** and symptom “B”—high severity **(B)** were analyzed by χ^2^ test. All the plants of each plot were considered to compare *Trichoderma* and control treatments. Where differences in distribution between the two treatments were significant, asterisks were placed (*P < 0.05, ^**^P < 0.01, and ^***^P < 0.001 significance levels, respectively).

The low level (A) of foliar symptom severity was the most frequent level of symptom in the first years of disease appearance in these young vineyards (Figure 5). The *Trichoderma* application reduced the incidence of the A symptom; this reduction was noticed in 92.6% of the year/vineyard combination and was statistically significant in 63% of the year/vineyard combinations. The V3-Trebbiano and V4-Cabernet Franc vineyards showed a slight increase of A symptoms in the treated plots only in the last year of application/investigation (Figure 5A), but this was not statistically significant. The incidence of severe symptom (B) was low, except in V4-Cabernet Franc where the severe symptoms were about 5% in 2017 and 2018. In such cases, however, symptom B was significantly lower (*P* < 0.001) in the treated plot (Figure 5B).

**FIGURE 6:**
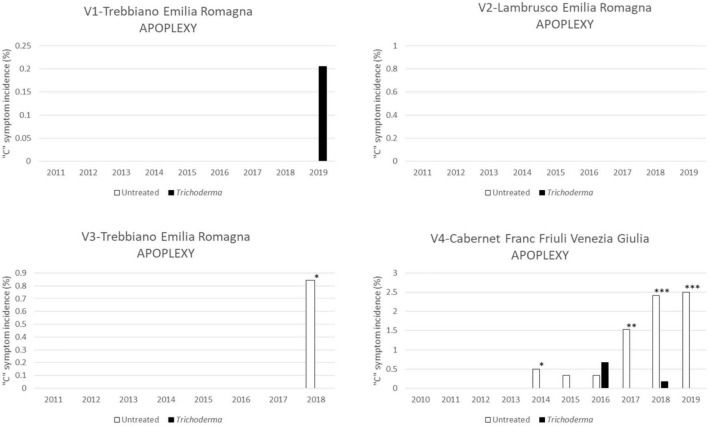
Apoplexy symptoms in the four young asymptomatic vineyards monitored for 8–10 years were analyzed by χ^2^ test. All the plants of each plot were considered to compare *Trichoderma* and control treatments. Where differences in distribution between the two treatments were significant, asterisks were placed (*P < 0.05, ^**^P < 0.01, and ^***^P < 0.001 significance levels, respectively).

The apoplectic symptom (Figure 6) was mainly observed in V2-Cabernet Franc with an incidence of <2.5%. In this vineyard, *Trichoderma* reduced the incidence of apoplexy in 5 out of 6 years (4 years with statistically significant differences).

**FIGURE 7:**
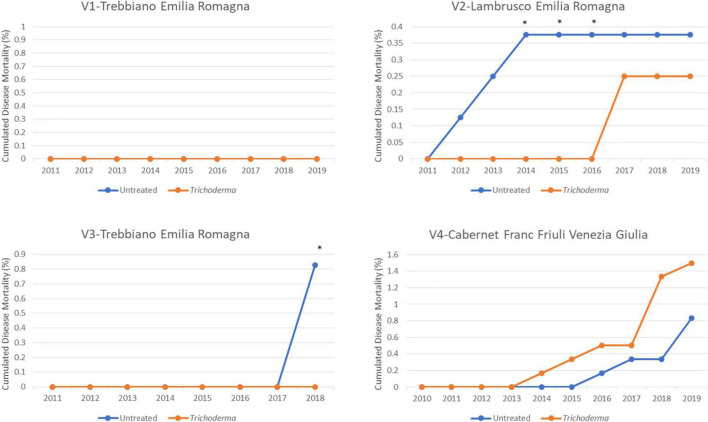
Cumulated mortality in the four young asymptomatic vineyards after 8–10 years of survey was analyzed by χ^2^ test. All the plants of each plot were considered to compare *Trichoderma* and control treatments. Where differences in distribution between the two treatments were significant, an asterisk was placed (*P* < 0.05).

Although death of the vines, as to be expected in such young vineyards, was absent (V1-Trebbiano) or lower than 1.5% in the other vineyards, the application of *Trichoderma* always reduced vine death, with significantly different values in 2 out of 3 vineyards (Figure 7).

**FIGURE 8:**
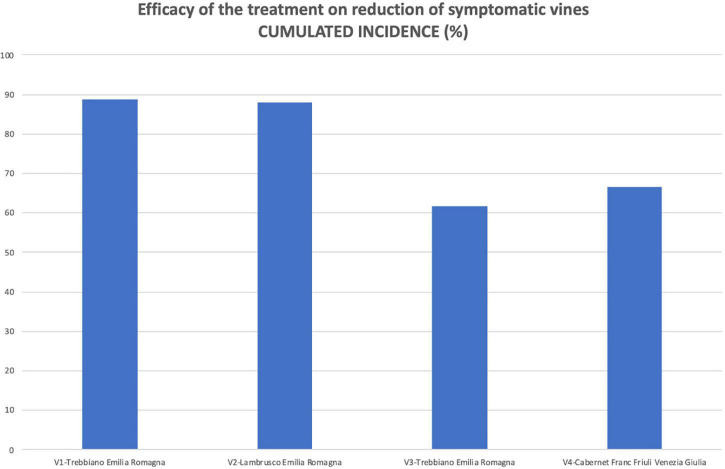
The efficacy of wound protection by the *Trichoderma* commercial product in reducing the number of symptomatic vines was calculated on the final number of vines that showed symptoms at least once during the whole survey time (cumulated incidence at the end of the trial).

As reported in Table 1, the number of vines surveyed in the two plots (treated and untreated) in each vineyard was identical in V2 and V4 vineyards and very close in V1 and V4. Therefore, as an overview of the efficacy of the protection of the pruning wounds by the *Trichoderma*-based product, a significant reduction in the symptoms given by the early protection of the wounds (Figure 8) was observed. The reduction in the number of vines that had been symptomatic at least once by the end of the 9-year surveys (cumulated incidence) divided by the total number of symptomatic vines in the two plots of that vineyard was ranging as follows: 88.7 (V1), 88.1 (V2), 71.7 (V3), and 61.7% (V4, which was the vineyard with the most susceptible cultivar).

## Discussion

This study is the first one carried out to test wound protection over a period of several years on several young vineyards that never showed GLSD foliar symptoms before. The study was carried out in commercial vineyards located in different Italian regions planted with different cultivars and showed that the protection activity of *Trichoderma* treatments applied on pruning wounds gives a remarkable reduction on the subsequent development of foliar symptoms and mortality associated with GLSD within the ECDs.

The study demonstrated the protective potential of *Trichoderma* applications against some of the associated GTD fungal pathogens *in vitro* and in artificial inoculation experiments. The *in vitro* trials with two single strains of *T. asperellum* and *T. gamsii* present in the commercial formulation applied in the field demonstrated that both strains inhibited or reduced, with different modes of action, the growth of selected GTD pathogens such as *Pch*, *Np*, and *Fmed*. Only *T. asperellum* ICC 012 showed direct activity as competitive growth.

Several studies have already shown that *T. asperellum* strains were effective against different pathogens through a combination of modes of action, such as antibiosis, mycoparasitism, competition for nutrients, volatile compounds, and induced resistance (Verma et al., 2007; Kott et al., 2015; Wu et al., 2017). The inhibitory efficacy of *T. asperellum* ICC 012 increased at higher temperatures, regardless of the pathogen species. This agrees with Qui et al. (2017) who observed that the activity of *T. asperellum* is favored by higher temperatures due to the increased production of conidia in a shorter time.

Although *T. gamsii* ICC 080 did not show any activity in competitive growth, it provided indirect activity, presumably due to volatile and non-volatile antibiotic compounds as described by Anees et al. (2010) and Chen et al. (2016). This isolate also showed efficacy at low temperatures. This strain of *T. gamsii* was originally classified as *Trichoderma viride*, which was found to include several cold-tolerant strains (Kredics et al., 2003). Furthermore, *T. gamsii* was indicated as being well suited for low-temperature environments (Rinu et al., 2014). The adaptability to act at low temperatures can be a relevant feature in pruning operations carried out in the winter season.

The registered product applied in the field trials includes both strains of the two species. The coexistence of different species of *Trichoderma* is conditioned by a variety of extracellular lytic enzymes and secondary metabolites produced by each strain (Cardoza et al., 2005). Carro-Huerga et al. (2021) isolated several species of *Trichoderma* spp. from the bark of grapevine and tested their biocontrol capacity. The authors obtained promising results on the use of combinations of some of the tested species, highlighting the importance of assessing their intra- and inter-specific compatibility for determining their possible combinations.

The re-isolation of the two species from inoculated canes during this study confirmed the ability of the strains to persistently colonize the wood, thus providing a long-lasting protection. Nevertheless, their efficiency was strongly influenced by the environment as the protection achieved from *Pch* infections on the most superficial section varied considerably in the 2 years, but in both years, it prevented the deeper colonization of the canes by the pathogen.

The field study offered a new view on the preventive action of these biocontrol agents when applied to fresh wounds by treating new vineyards in the first 1 or 2 years after planting before symptom development. The significant reduction in symptom development (foliar symptoms and apoplexy) recorded in the following years is a clear demonstration of the preventive activity of applications to the pruning wounds in commercial vineyards. It is also clearly demonstrated that preventive actions, as stated in Hills et al. (2016), are the only really rewarding approach to control GTDs.

In contrast to other trials, the efficacy of symptom reduction was clear since the first year in all four vineyards. The early efficacy in symptom reduction after treatment application may suggest a possible role of the infection in a new wound, close to the bud that will sprout in that year, in foliar disease expression, as also postulated by Larignon (2017). This hypothesis might be indirectly supported by the increasing reduction of symptom incidence over the years in *Trichoderma*-treated plots observed in the present study, assuming that the other factors associated with symptom expression remained unchanged in the treated and untreated plots. Therefore, although more research is required, it might not be excluded that new wound infections can reach the shoots developing from the buds close to that wound, having a higher probability to contribute to the formation of leaf symptoms. Anyway, pruning wound infections, well known as the most important point of entry of wood pathogens in the vineyard (Mugnai et al., 1999; Bertsch et al., 2013), remain of utmost importance for disease spread in vineyards.

The application of *Trichoderma* was carried out in March when the average daily mean temperatures were not lower than 10°C to favor wood colonization by the biocontrol agents. Temperatures can play an important role in the activity of *Trichoderma* species (Pertot et al., 2016; Martinez-Diz et al., 2021). Formulations based on *T. atroviride* I-1237 strain (biologically active at temperatures above 5°C) and *T. atroviride* SC1 (biologically active at temperatures equal to or higher than 10°C) showed good colonization when applied after pruning (Prodorutti et al., 2012; Mounier et al., 2016; Reis et al., 2017; Berbegal et al., 2020). As demonstrated by the *in vitro* assay, the presence of *T. gamsii* ICC 080 in the formulation can probably ensure some activity even at low temperatures, as it was shown to be biologically active at 10°C. Overall, temperatures above 10°C, even if not essential, favor the establishment and activity of different species of *Trichoderma* (Kredics et al., 2003). Furthermore, although it is necessary to consider the vineyard climate, environment, and cultural practices, it is also relevant to apply the product as soon as possible, i.e., on a fresh wound. Applications of *Trichoderma* carried out soon after pruning can increase the efficacy of wound protection (Mutawila et al., 2016).

The *Trichoderma* species used in this study showed the ability to effectively colonize wounds, very likely reducing the growth and viability of pathogens that reached the wound surface and providing long-term protection. In trials carried out with the same *Trichoderma* formulation for the protection of pruning wounds in symptomatic vineyards, the activation of host defense mechanisms [as had been described by Zehra et al. (2017)] promoted by the *Trichoderma* application was also hypothesized (Bigot et al., 2020). The activation of polyphenol biosynthesis (Patel and Saraf, 2017) with effects on *Pch* and other wood pathogens has previously been reported (Mutawila et al., 2011b).

The vineyards treated with *Trichoderma* since the first or second year of planting, when fully asymptomatic, clearly showed a large delay in the onset of leaf symptoms, which moreover occurred on a much smaller number of plants and with reduced severity.

Other trials carried out in older vineyards and started when the disease was already present report a reduction of 22% of “Esca symptoms” (Bigot et al., 2020) or 50% of GTD symptoms (Mounier et al., 2016). In the present trial, the cumulated incidence was reduced by 61.7–88.7% over the total number of vines showing the disease in the two plots in each vineyard. Young vineyards, usually not yet affected by the disease, are therefore the ideal target for the preventive protection of pruning wounds by *Trichoderma* toward infections that may occur every year through the pruning wounds. Furthermore, early application proved to be effective from the first year of symptom development, while Bigot et al. (2020) revealed that application of the same *Trichoderma* product on older vineyards resulted in significant effects on already symptomatic vineyards only from the third year of application. Nevertheless, even if applications before the first appearance of symptoms are so efficient, applications of *Trichoderma* may be recommended also in older symptomatic vineyards because the reduction in the number of symptomatic plants resulting from the treatment is linked to a reduction in yield losses, not only in terms of the number of productive vines but also in terms of quality losses as demonstrated by the correlation between leaf symptom and loss in quality of must and wine (Calzarano et al., 2004b; Lorrain et al., 2012).

A positive and increasing efficacy of *Trichoderma* applications in reducing apoplexy over the years could also be noticed, leading to the hypothesis that the reduction of annual infection might be correlated with a better plant defense reaction as suggested by Bigot et al. (2020). Biological control agents and *Trichoderma*, in particular, are known to show multiple effects that may have a role in the interaction between the host plant and the pathogen (Yacoub et al., 2016).

Finally, chemical fungicide formulations applied before (i.e., 24 h or a few days) the inoculation of pruning wounds with GTD pathogens proved to be effective in reducing infections (Brown et al., 2021; Martinez-Diz et al., 2021). The level of activity of these chemicals was superior to that of *Trichoderma* or *Bacillus amyloliquefaciens* formulations, and the authors commented on the need of a few days for growth and wound colonization and/or repeated applications of *B. amyloliquefaciens*. However, the ability of *Trichoderma* to get established and persist on pruning wounds for a long time on wounds that remain susceptible for months and against pathogens that produce new inoculum at every rain event is surely a competitive advantage.

In conclusion, the adaptability and versatility of *Trichoderma* species to several climatic and environmental conditions allow the fungus to display its activity under a wide range of climatic conditions (Hermosa et al., 2004), including those in which the grapevine is cultivated. Therefore, the protection of pruning wounds by *Trichoderma* can be likely recommended in all grapevine-growing areas. Moreover, the multiple interactions with the host plant proved to be effective for plant adaptation to climate change (Kashyap et al., 2017).

## Conclusion

Since the current management of the disease has limited control options (Mondello et al., 2018a), *Trichoderma* can be an effective tool for the reduction of both pruning wound infections and consequent wood colonization and degradation by fungal pathogens, of the expression of foliar symptoms in the vineyard, and of consequent losses in the quality and quantity of production. The efficiency of early and yearly protection soon after planting suggests the usefulness of applying efficient pruning wound protectants in mother vine fields, both for rootstock and scion vineyards, given the impact of GTD pathogens on nursery material.

Applications proved to be much more effective the earlier that they are carried out, that is, in young asymptomatic vineyards before the occurrence and spread of the disease. The treatment gives the highest efficacy after *Trichoderma* has established and grown on pruning wounds for a few days. Of course, the treatments need to be carried out every year on new wounds to protect the plants from new infections but, in this way, may also contribute to a reduction of the inoculum in the vineyard, which could be a useful integration with the recommended sanitary measures (Gramaje et al., 2018).

The disease reduction obtained by the early protection of pruning wounds by *Trichoderma* does not exclude further positive effects on the disease obtainable by other *Trichoderma* formulations as potential innovative formulations for curative trunk injection treatment (Peil et al., 2020).

Finally, it is important to remember that the *Trichoderma* species assessed in this study proved to be resistant to different pesticides used in viticulture, thus increasing the possibility to integrate chemical and biocontrol methods for the development of an effective and environmentally friendly control strategy for one of the most damaging grapevine trunk diseases worldwide.

This study demonstrated the relevance of pruning wound infections in causing leaf stripe symptoms (GLSD, with or without wood rot) and apoplexy within the Esca complex of diseases. It also confirms that multiple mechanisms and plant interactions are involved in the protective activity of *Trichoderma*, as stated by other authors (Druzhinina et al., 2011). Above all, it highlights the importance of early wound protection in maximizing the preventive activity and persistence of *Trichoderma* on annual infections and symptom expression of diseases in the Esca complex, such as leaf stripe and apoplexy.

## Data Availability Statement

The original contributions presented in the study are included in the article/supplementary material, further inquiries can be directed to the corresponding author/s.

## Author Contributions

SD and LM substantially conceptualized the study, designed the trials, acquired the data, and wrote the manuscript. EGM carried out the *in vitro* tests. SM and AP performed the data analysis. MN, GC, and FO did the *in vivo* inoculations and field surveys. FO and EB substantially contributed in discussion of the results and revision of the manuscript. All authors contributed to the article and approved the submitted version.

## Conflict of Interest

The authors declare that the research was conducted in the absence of any commercial or financial relationships that could be construed as a potential conflict of interest.

## Publisher’s Note

All claims expressed in this article are solely those of the authors and do not necessarily represent those of their affiliated organizations, or those of the publisher, the editors and the reviewers. Any product that may be evaluated in this article, or claim that may be made by its manufacturer, is not guaranteed or endorsed by the publisher.
